# Antibiotic stewardship in the PICU: Impact of ward rounds led by paediatric infectious diseases specialists on antibiotic consumption

**DOI:** 10.1038/s41598-020-65671-0

**Published:** 2020-06-01

**Authors:** Hanna Renk, Eva Sarmisak, Corinna Spott, Matthias Kumpf, Michael Hofbeck, Florian Hölzl

**Affiliations:** 1 University Children’s Hospital Tübingen, Dept. of Paediatric Cardiology, Pulmology and Intensive Care Medicine, Hoppe-Seyler Str. 1, 72076 Tübingen, Germany; 2 Department of Pharmacy, University Hospital Tübingen, Röntgenweg 9, 72076 Tübingen, Germany; 3 IT Project Management and Medical Controlling, University Hospital Tübingen, Geissweg 3, 72076 Tübingen, Germany; 4 Institute for Medical Microbiology and Hygiene, University Hospital Tübingen, Elfriede-Aulhorn-Straße 6, 72076 Tübingen, Germany

**Keywords:** Bacterial infection, Paediatric research

## Abstract

Antimicrobial stewardship programmes (ASP) are aimed at optimising antimicrobial utilization. However, only few studies have focused on paediatric intensive care units (PICU), where inappropriate antibiotic use occurs frequently. We assessed the effect and safety of a once weekly paediatric infectious disease (PID) ward round with prospective audit and feedback on antibiotic consumption in a multidisciplinary PICU. This study was conducted within 6-months periods before and after the implementation of a weekly PID-ward round. Antimicrobial management and two main recommendations per patient were discussed and documented. The primary outcome was antimicrobial utilization, measured by days of therapy (DoT) and length of therapy (LoT) per 1000 patient days (PD) for all PICU stays. Secondary outcomes included PICU mean length of stay, total mortality, infection-related mortality and cost of therapy. 1964 PD were analyzed during the pre- and 1866 PD during the post-implementation phase. Adherence to the recommendations was 79%. An 18% reduction of DoT/1000 PD was observed in the post-implementation period (p = 0.005). LoT/1000 PD decreased by 11% (p = 0.09). Meropenem and vancomycin usage were reduced by 49% (p = 0.07) and 56% (p = 0.03), respectively. We conclude, that a once weekly PID-ward round with prospective audit and feedback is safe and effective and reduces antibiotic consumption in PICUs.

## Introduction

Children admitted to the Paediatric Intensive Care Unit (PICU) represent a highly vulnerable patient population. Serious infections, both as a primary cause for admission and as a secondary healthcare-associated infection (HAI), are one of the most common problems encountered in a PICU^1^. Definite diagnosis of infection is often difficult and PICU clinicians are concerned about delayed treatment of infection. Therefore, empiric antimicrobial therapy is common practice in paediatric intensive care medicine. Antimicrobial utilization among PICU patients is as high as 51–76%^2–5^. Data suggest that as much as half of these treatments may be inappropriate and appropriateness of both empiric and definitive antimicrobial therapy remains a challenge^3,6,7^. The main reasons for antibiotic treatment in PICUs are perioperative antibiotic prophylaxis, suspected ventilator-associated pneumonia (VAP) and sepsis^3,7^. Moreover, 15–16% of PICU patients acquire HAI; patients after cardiac surgery are particularly at risk^8,9^. The most common antimicrobial agents used in PICU patients are 1st and 3rd generation cephalosporins, vancomycin and extended-spectrum penicillins. Notably, vancomycin is prescribed empirically in up to 50%^2,10^. Too long and not always well-founded utilization of reserve antibiotics is clearly correlated with selective pressure and the emergence of antibiotic resistances^11–13^. PICU clinicians face increasing rates of antibiotic resistances, along with highly sophisticated therapies of critically ill and frequently immunocompromised patients.

Antibiotic stewardship programmes have been developed to improve antibiotic management and utilization. Evidence from adult studies suggests that antibiotic stewardship interventions and collaborations between infectious disease and critical care practitioners can effectively improve antimicrobial utilization and patient outcome in intensive care medicine, including length of stay (LOS) and mortality^14,15^.

In the paediatric setting ABS is practical to implement as well and has no negative effects on patient safety^16^. Several antibiotic stewardship strategies have demonstrated promising results: (1) prospective audit and feedback, (2) formulary restriction and preauthorization and (3) structural interventions, e.g. broadening of inflammatory markers testing or screening methods^16^. Although appropriate use of antibiotics in a PICU is vitally important to ensure optimal clinical outcome neither the best method for, nor the effectiveness of antibiotic stewardship interventions in PICUs have been elucidated. Data on antibiotic stewardship prevalence in paediatric critical care medicine is extremely limited^3,17–21^.

The aim of this study was to assess the effect of a once weekly paediatric infectious disease ward round with prospective audit and feedback on antimicrobial use in a multidisciplinary PICU. We compared antibiotic consumption in a pre- and post-implementation period.

## Methods

### Study setting and population

The study was conducted at the closed 14-bed multidisciplinary PICU of the University Children’s Hospital Tübingen, Germany, which includes general paediatric patients, paediatric cardio-, general- and neurosurgical as well as haematooncological and solid organ transplant patients and offers an active Extracorporeal Life Support System (ECLS) program. The organizational structure of the ward remained unchanged for the study period. During the pre-implementation period in 2017, the PICU consultant in charge mainly directed antibiotic management. Written guidelines for perioperative antimicrobial prophylaxis and PICU empirical antibiotic guidelines were not available. The paediatric infectious disease team (PID-team) comprised a paediatric infectious disease specialist, a microbiologist, an infectious-diseases trained pharmacist and a virologist. The team implemented a weekly PID ward round three months prior to the start of the post-implementation period in 2018.

### Antibiotic stewardship intervention

The key feature of the antibiotic stewardship intervention was a once weekly infectious disease ward round using academic detailing and prospective audit with feedback focused on antimicrobial choice and consumption. The main goal of the intervention was an early de-escalation of broad empiric therapy guided by microbiological results, decreased utilization of antipseudomonal agents and increased use of a narrower penicillin-based antimicrobial therapy. The PID-specialist attended the PICU morning round once weekly to get insight into ongoing infectious disease management. History and course of disease, laboratory and microbiological findings, indications and antibiotic choice of all patients on antimicrobials were reviewed by the PID-team. Haemato-oncological patients were excluded. The same day, antimicrobial management of each patient was discussed with the PICU consultant and trainee in charge during the “30-minutes PID-ward round”. We defined seven possible recommendation groups (definitions are given in Online Resource 1). The two main antimicrobial stewardship recommendations for each patient were documented in the chart by the PID-specialist on the same day.

### Study design and outcomes

We conducted a prospective, pre- and post-implementation cohort study to assess the changes in antibiotic use during the six months of a once weekly PID-ward round on our PICU. The pre- and post-implementation periods were from January 2017 to June 2017 and January 2018 to June 2018, respectively. The primary outcome was antimicrobial utilization, as measured by critical-care unit-related antibiotic days of therapy (DoT) and length of therapy (LoT) per 1000 patient days (PD) for all antibacterial agents. Secondary outcomes included PICU mean length of stay, total mortality, infection-related mortality and cost of therapy. All research of this study was conducted in accordance with the Declaration of Helsinki and carried out in accordance with the guidelines of ICH-GCP. The study was approved by the local ethical review board at the University Hospital Tuebingen (project No 559/2019BO2) with a waiver of informed consent.

### Data collection and definitions

Demographic data, PICU length of stay, number of ECLS runs, mortality and infection-related mortality and Diagnosis Related Group (DRG)-relative weights were obtained from the hospital information system (i.s.h. med, SAP) by extraction of all PICU patients with one or more PICU stays of at least 24 hours in the study periods. Infection-related mortality was defined as any death that could be attributed to clinically or microbiologically confirmed infection as the immediate or underlying cause. Intensive care case mix index (ICU-CMI) was calculated by taking the sum of all DRG-relative weights divided by the total number of cases in the pre- and post-implementation period.

### Antimicrobial consumption data

Critical-care unit-related antibiotic use data were obtained using the antibiotic summary of each patient’s PICU stay from the clinical decision support and patient documentation system (IntelliSpace Critical Care and Anesthesia, Philips Healthcare). For each PICU stay of a patient of >24 h antibiotic consumption data were extracted into a separate Excel file. Re-admissions to the PICU were counted as separate visits if they were at least 24 h apart. The separate Excel files were aggregated via a custom Python script, computing days of therapy (DoT) and length of therapy (LoT) for each PICU stay^22,23^. DoT was defined as the number of days that a patient receives an antimicrobial agent, regardless of dose. All doses of a specific antibiotic administered on a given day counted as one DoT. The DoT for a given patient on multiple antibiotics was therefore the sum of DoT for each antibiotic the patient received. LoT accounted for the number of days that a patient received any antimicrobial agent, irrespective of the number of different antibiotics. Thus, LoT is always lower than or equal to DoT. For intergroup comparison DoT and LoT were standardized to 1000 patient days, using the PICU length of stay of each patient. To allow for detection of antibiotic consumption patterns and comparison of these data between hospitals, antibiotic density was calculated: DoT and LoT were aggregated and standardized to 1000 patient-days (DoT/1000 PD, LoT/1000 PD), using the total PDs in the respective period. We classified antibiotics according to the internationally standardized World Health Organization Anatomical Therapeutic Chemical (WHO ATC) classification system. Antibiotic costs were calculated for the median weight of the study population.

### Statistical analysis

Analysis was performed using Microsoft Excel 2013 and IBM SPSS Statistics Software Version 22 (Chicago, IL, USA). Descriptive statistics were done for baseline demographic and clinical characteristics of the pre- and post-implementation group. Categorical data was compared using the [chi]2 test. Fisher’s exact test was used in case of small expected observations. The nonparametric Mann–Whitney-U-Test was used for intergroup comparison of continuous variables that were not normally distributed. To assess statistical relevance of the change in antibiotic consumption between pre- and post-implementation period, we compared antibiotic density [DoT/1000PD] of single PICU stays, using Mann-Whitney-U-Test. A p-value < 0.05 was considered statistically significant. A detailed description of the statistical methods is provided in Online Resource 4.

## Results

Pre-implementation, 165 patients accounted for 183 PICU visits; post-implementation, 182 patients were addressed in 207 PICU visits. During the intervention, antibiotic stewardship recommendations were given for 38% (69 out of 182) of all patients. No statistically significant differences in age, gender distribution, weight or proportion of neonates were noted between the two periods. Distribution of departments of referral as well as the ICU-CMI and number of ECLS runs were similar (Table 1(b)).

**Table 1:** (a) Patients’ demographic characteristics and outcome in pre- and post-implementation period (cases analyzed n = 347).

(a)	Pre-implementation	Post-implementation	Difference	P value
**PICU cases analyzed**	183	207		
**Patient-days analyzed, days**	1964	1866		
**Number of patients**	165	182		
**Patients with ABS recommendations**	—	69 (38%)		
**Gender**				
male	88 (53)	103 (57)	+15	n.s.
female	77 (47)	79 (43)	+2	n.s.
Age, months*	8 [0–203]	11 [0–226]	+3	n.s.
neonates	39 (24)	33 (18)	-6	n.s.
Weight, kg*	7.2 [1.7–72.0]	8.2 [1.7–72.5]	+1.0	n.s.
**Department of referral**				
Cardiosurgical	75 (45)	64 (35)	−11	n.s.
General paediatric surgery	41 (25)	62 (34)	+21	n.s.
Neuropaediatrics and Neurosurgery	20 (12)	21 (12)	+1	n.s.
General paediatrics	29 (18)	35 (19)	+6	n.s.
**Outcome**				
Infection related mortality	1	2	+1	n.s.
Total mortality	4	9	+5	n.s.
**(b)**	**Pre-implementation**	**Post-implementation**	**Difference**	**P value**
ICU-CMI*	2.7 [0.2–55.8]	2.4 [0.3–36.4]	−0.3	n.s.
**ECLS runs**	5	5	0	n.s.
PICU LOS, days*	6 [2–112]	5 [2–77]	−1	n.s.
Ventilator-days*	1.6 [0–90]	1.6 [0–61.5]	0	n.s.

(b) PICU characteristics, PICU LOS and ventilator-days in pre- and post-implementation period (cases analyzed n = 390).

Data are numbers (%) or *median and [range]. Data of Table 1(a) and ECLS runs of Table 1(b) was compared using the [chi]2 test, Fisher’s exact test in case of small expected observations. Mann–Whitney-U-Test was used for ICU-CMI, PICU LOS and Ventilator-days. *p* < 0.05 was considered statistically significant.

**Figure 1:**
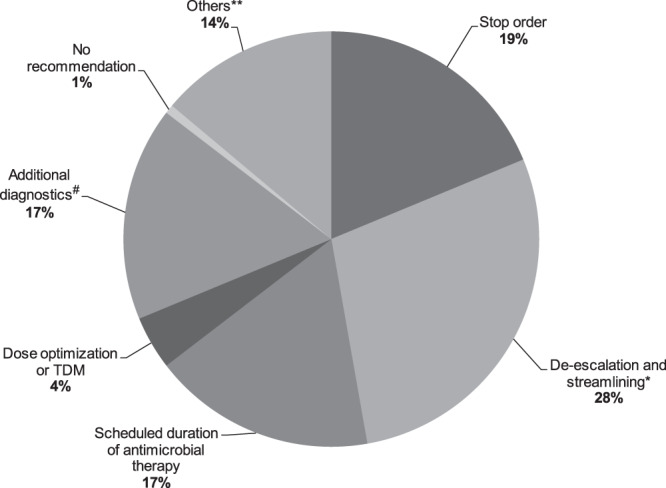
Overview of antibiotic stewardship recommendations. Frequency of the main antibiotic stewardship recommendations given during paediatric infectious disease ward rounds. *Using culture results as a basis for switching from broad-spectrum or multiple antimicrobials to more narrow-spectrum or targeted therapy; #e.g. microbiological or virological testing or repeated inflammatory markers etc; Others** - fixed established antibiotic regimens that could not be modified but were not in accordance with the recommendation given by the paediatric infectious diseases team.

In total, 104 antibiotic stewardship visits were carried out in 16 different PID ward rounds. Full or at least partial (one out of two recommendations followed) adherence to the recommendations by the PICU team was 79%. In 16 visits (15%) recommendations were not followed, and in 6 visits (6%) no recommendation was given. Almost half of all recommendations given were de-escalation and streamlining as well as stop orders (Fig. 1).

**Figure 2:**
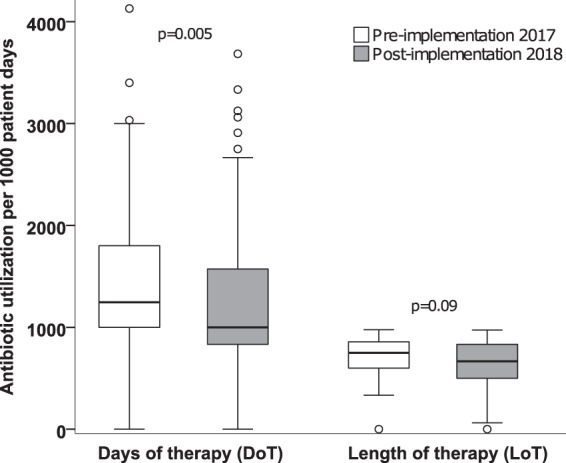
Total antibiotic use: Days of therapy (DoT) and length of therapy (LoT) per 1000 patient-days in the pre- and post-implementation period. Median [range] of days of therapy/1000 patient days, referring to all antibiotics used in the PICU, significantly declined by 18% in the post-implementation period (p = 0.005). Length of therapy/1000 patient days, referring to the days on any antibiotic therapy, declined by 11%. Mann-Whitney-U-Test, *p* < 0.05 was considered statistically significant. Error bars indicate minimum and maximum, circles indicate moderate outliers. DoT = Days of therapy, LoT = Length of therapy.

Overall, 1964 PD were analyzed during the pre-implementation and 1866 PD during the post-implementation phase. Total LoT was 1569 days before and 1333 days during the intervention. LoT/1000 PD was reduced by 83 days (p = 0.09). Total DoT decreased from 2959 days to 2431 in the post-implementation period. Accordingly, there was a significant reduction in total DoT/1000 PD of the PICU stays from 1226 [0–4129] to 1000 [0–3125] (p = 0.005), which represents a median difference of 18% between pre- and the post-implementation periods (Fig. 2, additional data are given in Online Resource 2). Secondary outcome data comprised PICU length of stay, ventilator-days, total mortality and infection-related mortality and were not affected by the antibiotic stewardship intervention. The mean PICU length of stay in the pre-implementation and post-implementation period was 6 [2–112] and 5 days [2–77], respectively. Mean duration of ventilation was 1.6 days in both periods. Due to an increase in total mortality in the post-implementation period, the proportion of infection-related mortality decreased from 25% to 22%, but the absolute number of infection-related deaths was not significantly different (Table 1(a)). Of note, the infection-related deaths in the post-implementation period were related to fungal sepsis and influenza pneumonia.

**Figure 3:**
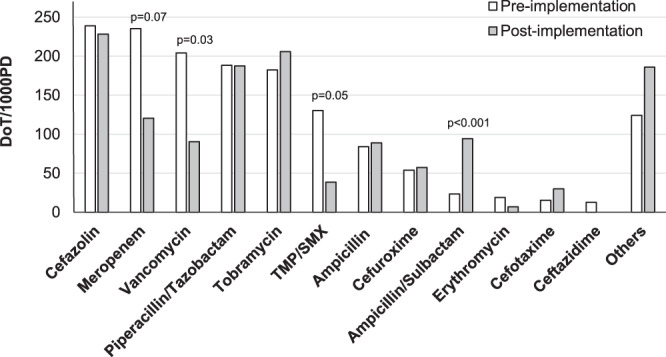
Pattern of PICU antibiotic utilization in the pre- and post-implementation period. Use of WHO “Watch group” antibiotics meropenem and vancomycin was halfed (meropenem by 49%, vancomycin by 56%), and use of trimethoprime-sulfamethoxazole decreased significantly during the post-implementation period (p = 0.05). As intended by the antibiotic stewardship team, a sharp increase in ampicillin/sulbactam utilization was observed in the post-implementation period. Mann-Whitney-U-Test was used for comparison of DoT/1000 PD for each antibiotic per PICU stay; *p* < 0.05 was considered statistically significant.

Antimicrobial consumption was analyzed in detail in the pre-implementation and the post-implementation period (Fig. 3, additional data are given in Online Resource 3). Antibiotic density measured as DoT/1000 PD decreased by 204 (14%) and was 1303 after the implementation. First-generation cephalosporins (mainly cefazolin) were by far the most frequently used antibiotics measured as absolute DoT and as antibiotic density with 257.1 DoT/1000 PD in the pre-implementation and with 228.3 DoT/1000 PD in the post-implementation period (p = 0.22). Utilization of penicillin/beta-lactamase inhibitor combinations increased by 34% (p = 0.02), largely influenced by a sharp increase in ampicillin/sulbactam utilization, while broad-spectrum piperacillin/tazobactam DoT remained stable. Anti-pseudomonal cephalosporin utilization significantly decreased to zero in the post-implementation period (p = 0.03). Carbapenems were the second most commonly used antibiotic class pre-implementation. Mean carbapenem utilization decreased by 44%, particularly for meropenem by 49%, which was marginally significant (p = 0.07). Consumption of aminoglycosides remained unchanged (p = 0.14). Use of glycopeptides, mainly vancomycin, significantly declined by 57% following initiation of the antimicrobial stewardship intervention (p = 0.02). A significant reduction was also observed in the consumption of trimethoprime/sulfamethoxazole with a decline of about 70% (p = 0.05). Utilization of all other antimicrobial substances remained largely unchanged.

Although not considered a primary objective, antibiotic costs were reduced from 4252€ to 3141€, a relative reduction of 26% with savings of 566€ per 1000 PD. Assuming full bed occupancy, this corresponds to a cost reduction of 2892€/year for antibiotics in our PICU.

## Discussion

Judicious and optimal antibiotic treatment in PICU patients is essential in light of the approaching “post-antibiotic era” with even fewer antibiotic options for children compared to adults^23,24^. To the best of our knowledge, this is the first study that evaluated DoT/1000 PD of the total antibiotic consumption in detail along with LoT/1000 PD in a multidisciplinary PICU before and during weekly PID-ward rounds with prospective audit and feedback. Prospective audit and feedback has previously been proposed as an antibiotic stewardship intervention in adults and children^24–26^. However, the impact of this type of intervention in the PICU setting is not known, with only a limited number of published reports related to PICUs^3,17–21^. In this study, complete or partial adherence (at least one out of two recommendations followed) to the given antibiotic stewardship recommendations was 79%. Previous paediatric ASPs have reported comparable adherence of 77–89%^27–30^. In 15% ASP recommendations were not followed, mainly because of non-compliance of PICU physicians who felt, that the patient was “too sick” for a narrow-spectrum therapy, or a sudden deterioration of the patient hampered a “step-down therapy”.Sometimes a change in renal function or additional results required other therapeutic adaptions than previously recommended.

In a point prevalence survey in a similar setting to ours, the main causes of inappropriate use of antibiotics were overly broad spectrum, wrong dosage and unwarranted overlap of spectra^3^. In our study, the majority of recommendations were also related to a reduction of antibiotic utilization with 64% being either de-escalation and/or streamlining, scheduled duration of therapy or stop order.

Although, we only reached 38% of all patients with a once weekly PID ward round, we found a significant reduction in median antibiotic utilization (p = 0.005). Antibiotic density was 1507 DoT/1000 PD before the antibiotic stewardship intervention. We cannot state, whether this is a high or low rate of antibiotic consumption, since numbers of studies in comparable settings are scarce. Lee *et al*. reported a total DoT/1000 PD of 1201 before an antibiotic stewardship intervention in the US, which is more or less comparable to ours^31^. Taking this study as a reference, the antibiotic consumption before and after the implementation was slightly higher in our setting. Antibiotic density measured as DoT/1000 PD decreased by 204 (14%) and was 1303 after the implementation. Others reported similar reductions, largely depending on the setting and baseline antibiotic consumption. A systematic review of the impact of antimicrobial stewardship in critical care in adult populations found reductions of antimicrobial utilization of 11–38% in 24 studies^14^. Willis *et al*. and Newland *et al*. both reported a comparable decrease in overall antibiotic DoT/1000 PD by 11% after the implementation of a Prospective-Audit-With Feedback Antimicrobial Stewardship Program at a Children’s Hospital, not limited to the PICU^24,27^. In other PICU settings reductions of 21–64% were described^18,31^. In summary, our result seems reasonable when compared to the few studies in similar settings and with similar paediatric antibiotic stewardship interventions.

Evaluation of the absolute length of antibiotic treatment is important, since the prescription of broad-spectrum antibiotics reduces DoT, whereas the combination of several narrow spectrum antibiotics increases it. LoT is not influenced by this phenomenon and therefore provides additional information about antibiotic utilization^32^. Mean LoT/1000 PD decreased by 83.3 (11%) during the implementation, although this reduction did not reach significance. Since no other PICU studies have evaluated LoT/1000 PD before and during antibiotic stewardship interventions, we cannot compare our data with other PICUs. Other children’s hospitals showed significant decreases of LoT/1000 PD by 7–8% after similar antibiotic stewardship interventions, based on larger cohorts with more than twice the amount of patients and patient days compared to our study^24,33^. A prolonged study period with a larger number of cases and patient days may be necessary to further investigate potential reductions of LoT/1000 PD in the PICU setting.

Detailed analysis of single agents and antibiotic classes revealed a remarkable decrease in DoT/1000 PD of antimicrobials targeted by the antibiotic stewardship intervention. Cefazolin was the most commonly used single agent before and after the antibiotic stewardship intervention. In line with our findings, Blinova *et al*. found cefazolin, cefuroxime, vancomycin and gentamicin as the most commonly used antibiotics in his point prevalence survey in a cardiac and medical-surgical paediatric critical care unit^3^. Cefazolin is used as perioperative prophylaxis in cardiac surgery and the high antibiotic consumption of this agent in our study is explained by a postoperative regimen that favours prolongation of prophylaxis until all medical devices including Central Venous Catheters (CVCs) are removed.

Meropenem was the second most frequently used antibiotic before the implementation and accounted for 16% of all antibiotics prescribed. DoT/1000 PD of meropenem had been reduced by almost 50% and accounted only for 9% of antibiotics used in the post-implementation period (p = 0.07). Two approaches primarily contributed to the reduction in antibiotic density of meropenem: First, through discussions during the PID-ward round, we tried to switch to penicillin/beta-lactamase inhibitor combinations (e. g. ampicillin/sulbactam or piperacillin/tazobactam) whenever there was no proof of an Extended-Spectrum ß-lactamase (ESBL) or multidrug-resistant pathogen. Second, antibiotic time-out and microbiological diagnostics after 24 h to 48hrs off antibiotics were recommended, if meropenem therapy did not have any effect after 10–14 days of treatment. Thereby, long-term carbapenem courses of 3–4 weeks were avoided. Recently, Hoshina *et al*. have also shown a reduction of carbapenem consumption in paediatrics through consultation of a paediatric infectious disease specialist. Notably, in the 5 year study period, the proportion of carbapenem utilization decreased from 13% to 4.3% and the incidence of meropenem sensitive *Pseudomonas aeruginosa* significantly increased^34^.

Appropriate prescription of glycopeptide antibiotics was a main target of the antibiotic stewardship intervention, as in previous paediatric studies^35,36^. Antibiotic density was significantly reduced by 57% from the pre- to the post-implementation period (p = 0.02). Two other studies in the PICU setting indicate a decrease of vancomycin utilization of 35% and 50% respectively^18,31^. The significant reduction in our study is explained by several factors: First, vancomycin utilization was one of the main targets of our antibiotic stewardship intervention. Second, excessive use due to an empiric escalation regimen in the pre-implementation period was high. Recommendations for vancomycin use during the post-implementation period were based on Methicillin-resistant *Staphylococcus aureus* (MRSA) screening results and probability of catheter-associated blood stream infection due to resistant coagulase-negative staphylococci or the risk of severe *Enterococcus faecium* infection. Last, we only observe a very low rate of MRSA colonization in our setting.

Cotrimoxazole utilization decreased significantly by 70% (p = 0.05). This trend remains rather unclear to us and we can only speculate, that discussion during the PID ward round about indications of antimicrobials may also have influenced the prescription of cotrimoxazole. In contrast to the overall reduction in antibiotic utilization, we observed a significant increase in DoT/1000 PD of ampicillin/sulbactam, from 2% to 7% of all antibiotics prescribed (p < 0.001) in the post-implementation period. This increase was intended by the PID team, since it is well known that penicillin-based therapy has less influence on the intestinal microbiota, causes less selective pressure than broad-spectrum cephalosporins or carbapenems and is therefore classified among the key “ACCESS” antibiotics in the WHO Model List of Essential Medicines for Children^37–39^.

Disease severity measured by the ICU-CMI was similar before and during the implementation. To ensure the antibiotic stewardship recommendations did not cause harm, we determined secondary outcome measures including PICU length of stay, ventilator-days, and total hospital mortality. In particular, infection-related mortality remained stable between the study periods. Therefore, we conclude that the antibiotic stewardship intervention with significant reduction of overall DoT/1000 PD is safe.

As many other antibiotic stewardship studies, our study is limited to a quasi-experimental pre- and post-implementation design and limited to a single centre. However, we did compare the same seasonal periods and no major changes of the patient or staff structure of the PICU that could have affected antibiotic prescribing practices occurred between the study periods. DoT/1000 PD is a widely accepted measure for antimicrobial use in paediatrics, since it is independent of a patient’s weight, but cannot detect breadth, toxicity and costs of antimicrobial prescription. Furthermore, in this analysis we could not assess the effect of the intervention with respect to patient-related outcomes. As an indirect measure, we used secondary indicators of antibiotic treatment failure - including PICU LOS, ventilator-days and infection related mortality - although they might not be particularly sensitive.

## Conclusion

In conclusion, this study demonstrates that an ASP with prospective audit and feedback during once weekly ward rounds led by paediatric infectious diseases specialists is associated with good adherence to antibiotic stewardship recommendations. This intervention could significantly reduce antibiotic consumption in a multidisciplinary PICU without negatively affecting patients’ safety. Generalizability, sustainability and clinical outcomes of PICU antimicrobial stewardship need further elucidation.

## Supplementary information

Supplementary Information.

Supplementary Information2.

## Data Availability

Most data analyzed during this study are included in this published article (and its Online Resources 1–4). The underlying datasets generated during the current study are available from the corresponding author on reasonable request.
