# Supplementary material for: Antibiotic stewardship in the PICU: Impact of ward rounds led by paediatric infectious diseases specialists on antibiotic consumption
Source: Sci Rep. 2020 Jun 1;10:8826. doi: 10.1038/s41598-020-65671-0 (PMC7264238; doi:10.1038/s41598-020-65671-0)
Supplement: Supplementary file 1 — Supplementary Information. [file 41598_2020_65671_MOESM1_ESM.pdf]

Antibiotic stewardship in the PICU: Impact of ward rounds led by paediatric infectious diseases specialists on antibiotic consumption

Authors: Hanna Renk[1*], Eva Sarmisak[2], Corinna Spott[3], Matthias Kumpf[1], Michael Hofbeck[1], Florian Hölzl[4]

[1]University Children's Hospital Tübingen, Dept. of Paediatric Cardiology, Pulmology and Intensive Care Medicine, Hoppe-Seyler Str. 1, 72076 Tübingen, Germany.

[2]Department of Pharmacy, University Hospital Tübingen, Röntgenweg 9, 72076 Tübingen, Germany.

[3]IT Project Management and Medical Controlling, University Hospital Tübingen, Geissweg 3, 72076 Tübingen, Germany.

[4]Institute for Medical Microbiology and Hygiene, University Hospital Tübingen, Elfriede-Aulhorn-Straße 6, 72076, Tübingen, Germany.

Online Resource 1

Types of antibiotic stewardship recommendations

(1) De-escalation and streamlining: Tailoring antibiotic therapy by using culture results to switch from broad-spectrum or multiple antimicrobials to more narrow-spectrum or targeted therapy.

(2) Dose optimization or Therapeutic Drug Monitoring (TDM)

(3) Stop order

(4) Additional diagnostics included microbiological or virological testing or repeated analysis of inflammatory markers.

(5) Scheduled duration of antimicrobial therapy

(6) Others summarized situations in which the PID team accepted but did not agree with the established antibiotic regimen for perioperative antibiotic management of a patient subgroup.

(7) No recommendation

Online Resource 4

Detailed description of data analysis and statistical methods:

1.) Calculation of DoT and LoT:

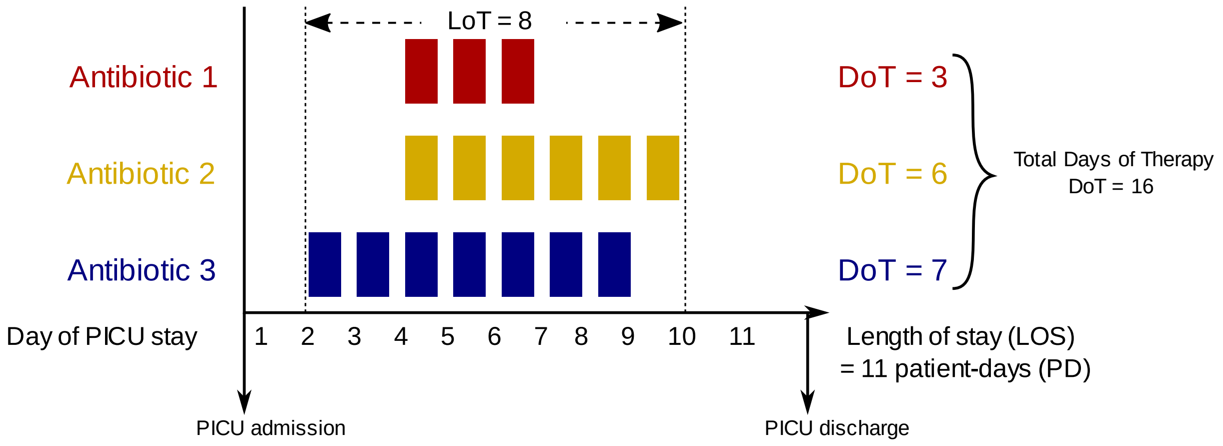

To standardize DoT and LoT for the length of PICU stay, we calculated DoT/1000 PD and LoT/1000 PD for each PICU stay in the pre- and postimplementation period as follows:

DoT/1000 PD: *1000*(Total DoT of a single PICU stay / length of stay of this single patient on PICU)*

LoT/1000 PD: *1000*(Total LoT of a single PICU stay / Patient days of a single patient on PICU)*

2.) Data structure:

We extracted the above mentioned data from the patient records. Calculations resulted in the following data structure (only two antibiotics and two patients are given as an example):

ID	Group pre(1) post(2) implementation	Length of Stay in PICU (PD)	LoT	LoT/1000PD	Total DoT	Total DoT/1000PD	DOT Penicillin	DOT/1000d Penicillin	DOT Gentamicin	DOT/1000d Gentamicin
P1	1	4	4	1000	7	1750	3	750	4	1000
P2	2	8	5	1875	5	1875	5	625	0	0
….										

3.) Data analysis

Part 1:

Data of the pre-intervention period were compared to data of the postintervention period by Mann-Whitney U-Test. $p<0.05$ was considered statistically significant. The following data of single PICU stays were compared: LoT/1000 PD, DoT/1000 PD and DoT/1000 PD of every antibiotic administered.

Part 2:

In a second step, the pattern of antibiotic distribution (antibiotic density) was analyzed for the pre- and postintervention period.

Total DoT and DoT for every antibiotic of all PICU stays in the pre- and postintervention period were aggregated as well as Length of stay (patient-days) for both periods. Subsequently, antibiotic density was calculated as follows:

Antibiotic density for all antibiotics:

DoT/1000 PD: *1000*(Total DoT of all PICU stays / total patient-days in the respective period)*

Antibiotic density for every single antibiotic (e.g. Penicillin):

DoT/1000 PD: *1000*(Total Penicillin DoT of all PICU stays / total patient-days in the respective period)*

Antibiotic density data of every antibiotic reflect the antibiotic utilization pattern of the total ward in the pre- and postimplementation period. Statistical significance of the differences in utilization were determined by intergroup comparison of DoT/1000 PD related to length of stay of a single PICU stay as described in section Part 1.
